# Wound Healing Activity of *Elaeis guineensis* Leaf Extract Ointment

**DOI:** 10.3390/ijms13010336

**Published:** 2011-12-28

**Authors:** Sreenivasan Sasidharan, Selvarasoo Logeswaran, Lachimanan Yoga Latha

**Affiliations:** 1 Institute for Research in Molecular Medicine (INFORMM), Universiti Sains Malaysia, 11800 USM, Pulau Pinang, Malaysia; 2 Department of Biotechnology, Faculty of Applied Sciences, Asian Institute of Medicine, Science and Technology, 08000 Sungai Petani, Kedah, Malaysia

**Keywords:** *Elaeis guineensis*, wound healing activity, matrix metalloproteinases, gelatin zymography

## Abstract

*Elaeis guineensis* of the Arecaceae family is widely used in the traditional medicine of societies in West Africa for treating various ailments. To validate the ethnotherapeutic claims of the plant in skin diseases, wound healing activity was studied. The results showed that *E. guineensis* leaf extract had potent wound healing capacity as evident from the better wound closure (*P* < 0.05), improved tissue regeneration at the wound site, and supporting histopathological parameters pertaining to wound healing. Matrix metalloproteinases expression correlated well with the results thus confirming efficacy of *E. guineensis* in the treatment of the wound. *E. guineensis* accelerated wound healing in rats, thus supporting its traditional use. The result of this study suggested that, used efficiently, oil palm leaf extract is a renewable resource with wound healing properties.

## 1. Introduction

Malaysia is the largest producer of oil palm (*Elaeis guineensis* Jacq.) in the world and producing more than 7 Million tons of crude palm oil. The total planted area of oil palm increased from 73,000, reaching 3.87 million hectares in 2004 [1]. Oil palm frond (OPF) contributes 70% of the overall oil palm industry waste in Malaysia [2]. It is reported that Malaysia alone produced about 30 million tons annually of oil-palm biomass, including trunks, fronds, and empty fruit bunches [3]. One of the significant problems in the palm fruit processing is managing the wastes generated during the processes. In Malaysia, for example, 9.9 million tons of solid wastes consisting of oil-palm empty bunch, fiber and fruit shell and 10 million tons of palm oil mill effluent (POME) are generated every year. In Malaysia, a 3.87 million ha area is used for plantation of oil palm and large quantities of cellulosic and non-cellulosic raw material are generated during harvesting [1,4]. The expansion of plantation in this country has generated large amounts of agro waste, creating problems in replanting operations and tremendous environmental concerns. When left on the plantation floor, these waste materials create great environmental problems [5,6].

In traditional medicine, the leaf of *E. guineensis* is squeezed and the juice that is obtained is placed on wounds to promote healing [7]. The sap of this plant is also used as a laxative and the partially fermented palm wine is administered to nursing mothers to improve lactation. Soap prepared with ash from fruit-husk is used for the preparation of a soap used for skin infections. A root decoction is used in Nigeria for headaches. The pulverized roots are added to drinks for gonorrhea, menorrhagia and as a cure for bronchitis [7]. The fruit mesocarp oil and palm kernel oil are administered as poison antidote and used externally with several other herbs as lotion for skin diseases. Palm kernel oil is applied to convulsant children to regulate their body temperature. Folk remedies of oil palm include treatment for cancer, headache and rheumatism and as an aphrodisiac, diuretic and liniment [7]. The antibacterial activity of this plant extract against different micro-organisms and antioxidant activity have already been reported [8,9]. Moreover, Sasidharan *et al*. [10] described the potential of *E. guineensis* leaf methanol extract as an infected wound healing agents. They observed that the bacterial count in the *E. guineensis* extract treated rats was significantly reduced to 10^2^ CFU/g tissue on day 16. In this study, we tested wound healing activity without infection and the expression of Matrix metalloproteinases. The recent study also reported the standardization of *E. guineensis* leaf methanol extract with respect to authenticity, assay and chemical constituent analysis by Rajoo *et al*. [11]. Syahmi *et al*. [12] also tested the toxicity of *E. guineensis* leaf methanol extract against brine shrimp (*A. salina*) and mice. The results of both tests confirmed that *E. guineensis* is nontoxic and they recommended it as a safe natural product for commercial utilization. Based on its use in wound healing in traditional practices and literature references, the present study was undertaken to evaluate the wound-healing activity of *E. guineensis* and is reported hereunder.

Wounds are physical injuries that result in an opening of the skin. Proper healing of wounds is essential for the restoration of disrupted anatomical continuity and disturbed functional status of the skin. Many researchers have reported the enhancement in the wound healing process by various plant extracts and isolated compounds in animal models in literature. However, the exact mechanism involved in the wound healing process is still not clear. Moreover, it is a fact that there are number of parameters which are involved in the healing of wound including epithelialization, antioxidant defense, estimation of hydroxyproline content in the granulation tissue, biochemical changes and *in silico* validation. Hence, in this study we are reporting wound closer rates, wound contraction, epithelialization and matrix metalloproteinases (MMPs) expression. The use of *in silico* computational models holds the promise to improve the understanding of the process of wound healing. By modifying an existing ordinary differential equation model of systemic inflammation to simulate local wound healing, the understanding of the underlying complexities of wound healing will be improved, thus allowing for the development of novel, targeted therapeutic strategies [13]. The use of *in silico* computational models should be the approach for the future studies on wound healing activities of *E. guineensis* leaf extract.

## 2. Results and Discussion

### 2.1. Wound Closure Rate

**Figure 1:**
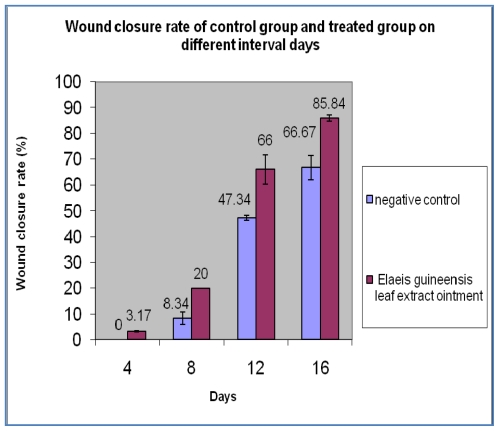
Percentage wound closure in the control and treated groups in different time intervals (*n =* 8; *P <* 0.05). Results are presented as mean ± SD.

**Figure 2:**
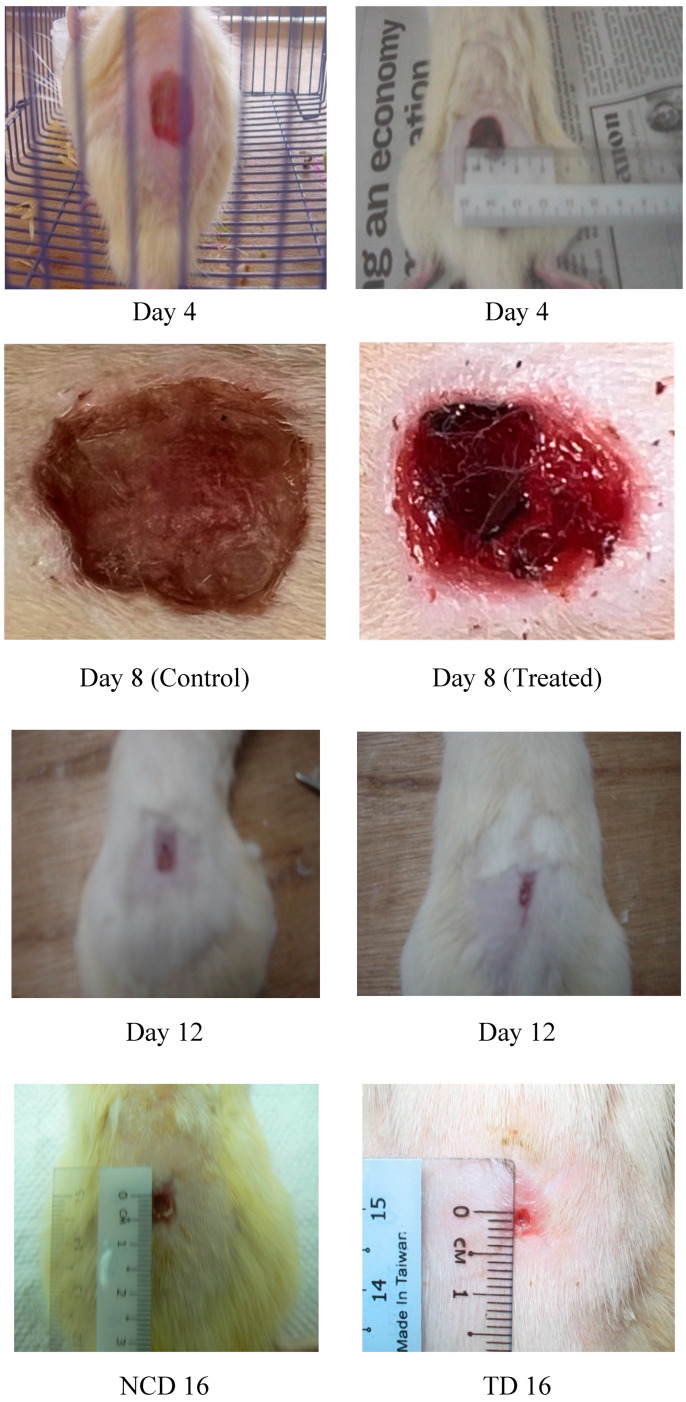
Photographical representation of contraction rate on different days in control group.

Significant difference in the wound closure was observed in treated group from day 4 onwards and also the rate of wound closure was much faster on later days when compared with control. Complete wound closure was observed in *E. guineensis* leaf extract treated group on day 16 whereas in control group it was about 25 days (Figures 1 and 2).

### 2.2. Histopathology Analysis

**Figure 3:**
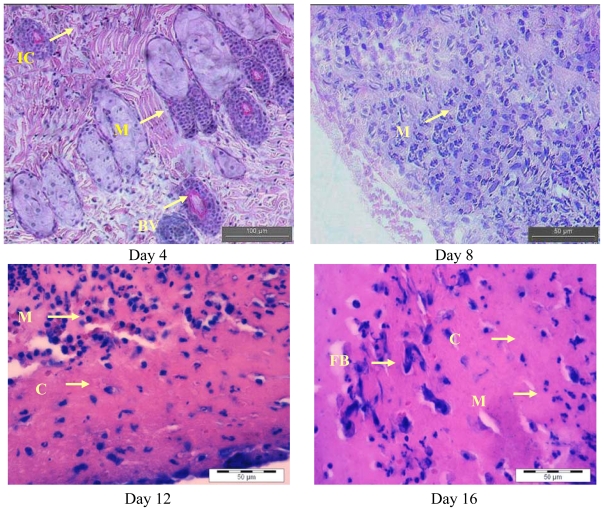
Hematoxylin and eosin stained sections of the granulation tissue in control group at different time intervals. Fibroblasts (FB), macrophages (M), collagen (C) bundles, Vascularization with larger blood vessels (BV) and inflammatory cells (IC).

**Figure 4:**
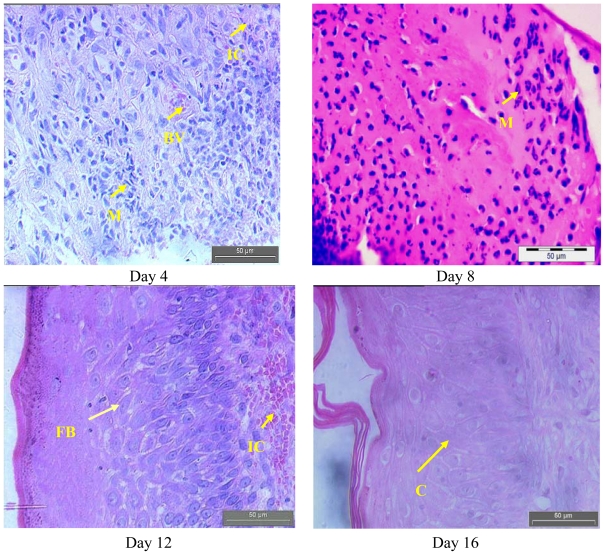
Hematoxylin and eosin stained sections of the granulation tissue in treated group at different time intervals. Fibroblasts (FB), macrophages (M), collagen (C) bundles, Vascularization with larger blood vessels (BV) and inflammatory cells (IC).

Figures 3 and 4 show the histological analysis of granulated tissue from control and treated groups on different days. The histological study of granulation tissue of control (Figure 3) animals demonstrated a more aggregation of macrophages with few collagen fibers than the treated groups (Figure 4). The animals treated with the methanol extract exhibited a significant increase in collagen deposition with fewer macrophages and more fibroblasts. A significant increase in collagen content was observed during the wound healing process in the treated group resulted due to enhanced migration of fibroblasts and epithelial cells to the wound site. Moreover, as shown by previous studies, oil palm contains ascorbic acid, which acts as a cofactor for the synthesis of collagen as well as elastin fibers [14]. The decreased collagen content in the control group might be due to prolonged inflammatory phase where the degradation of collagen will be more than the synthesis collagen. Furthermore, collagen is a major protein of the extracellular matrix and is the component that ultimately contributes to wound strength. The breakdown of collagen liberates free hydroxyproline and its peptides. Measurement of the hydroxyproline could be used as an index for collagen turnover. Therefore, the increment of hydroxyproline content in the animals treated with plant extract indicating increased collagen turnover [15]. In a future study we intended to investigate the hydroxyproline content in *E. guineensis* leaf extract treated group.

It is also evident that the antioxidant supplementation helps to reduce the level of oxidative stress and to slow or prevent the development of complications associated with diseases [16]. In addition, the reactive oxygen species (ROS) are deleterious to wound healing process due to the harmful effects on cells and tissues. The granulation tissue consisting of new capillaries and fibroblast may be replaced by hematoma within the wound. Plant extracts are potential wound healing agents, and largely preferred because of their widespread availability, non-toxicity. absence of unwanted side effects, and effectiveness as crude preparations [10]. Previously, it was reported that *Carapa guienensis, Carica papaya* and *Jasminum grandiflorum* extracts are effective in wound healing in rats [17–19]. In this study, we also have clearly observed an enhanced wound contraction induced by the *E. guineensis* leaf extract. This could be attributed to the enhanced contractile property of myofibroblast resulting in the increase of epithelialization. Thiem and Goslinska [20] have reported that topical application of compounds with free radical scavenging properties in patients have shown to improve wound healing significantly and protect tissues from oxidative damage. Our previous study showed that the *E. guineensis* leaf extract possessed free radical scavenging property [8]. Hence, this could contribute the wound healing activity observed in this study.

A close examination of granulation tissue sections revealed that the tissue regeneration was much quicker in the treated group compared to control wounds (Figures 3 and 4). Early dermal and epidermal regeneration in the treated group confirmed that the ointment containing *E. guineensis* extract had a positive effect toward cellular proliferation, granulation tissue formation, and epithelialization. Well-formed collagen bundles in the treated group shown in hematoxylin and eosin staining support the efficacy of *E. guineensis* on fibroblast proliferation and synthesis of extracellular matrix during healing (Figure 4). Incomplete epithelialization with less extracellular matrix synthesis was observed in control rats as can be seen in Figure 3. Clumps of degenerating neutrophils, necrotic changes, and the persistence of inflammatory exudates in the upper dermis with loss of epidermis were also observed up to day 16. Treated rats have shown marked epithelialization, moderate amount of extracellular matrix synthesis and new blood vessel formation. Our previous phytochemical analysis results revealed the presence of tannins, alkaloids, reducing sugars, steroids, saponins, terpenoid, and flavonoids in the methanolic extract [10]. The constituents of the oil palm leaf extract, such as terpenoids and alkaloids, may play a major role in the wound healing process observed in this study.

### 2.3. Gelatin Zymography

**Figure 5:**
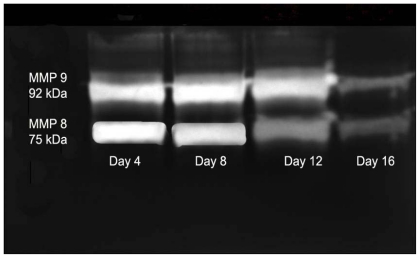
Zymogram shows the expression of matrix metalloproteinases (MMPs) in *Elaeis guineensis* leaf extract ointment treated group.

**Figure 6:**
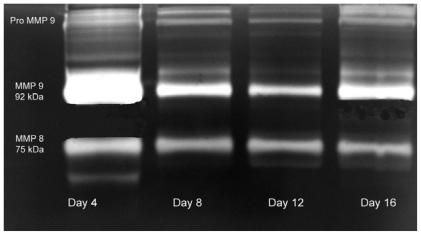
Zymogram shows the expression of MMPs in negative control rat group.

Gelatin zymography analysis shows the expression of pro and active forms of MMPs in the granulated tissue from the treated rats (Figure 5) at different days of healing (days 4, 8, 12 and 16). In the initial days of healing the expression of polymorphonuclear eutrophils derived MMPs (MMP 8–75 kDa, MMP 9–92 kDa) were high followed by a reduction in later days of healing. Expression of these MMPs was more in control rats (Figure 6) and persists until day 12.

The expression of MMPs revealed the enhanced healing pattern in the *E. guineensis* leaf extract treated rats group compared to control group. MMP 8, and MMP 9 are the major MMPs observed in the present study. These MMPs also facilitate fibrin and eschar removal and the resultant peptides are known to possess chemotactic and angiogenic properties [21]. Neutrophil-derived matrix MMP-8 is the predominant collagenase present in normal healing wounds, and over expression and activation of this collagenase may be involved in the pathogenesis of non-healing chronic leg ulcers [22]. We have observed the expression of MMP 8 during the twelve days. It reduced sharply in later days, which shows the enhanced healing by the *E. guineensis* leaf extract treatment. Expression of MMP 9 on early days suggests that it might have involved in keratinocytes migration and granulation tissue remodeling [23]. However, we observed that the expression of MMP 9 was sharply reduced in later days.

## 3. Experimental Section

### 3.1. Sample Collection

Five kilograms of fresh leaf of *E. guineensis* were collected from A.V.B. oil palm Estate Semeling, Bedong, Kedah, Malaysia, in June 2008. Voucher specimens (Voucher number of 11036) were retained in our laboratory for further reference. The leaf was separated and cut into small pieces. The small pieces of the leaf were washed with tap water followed by distilled water. The fresh leaf material was then dried in an oven at 60 °C for 7 days. The dried leaf of *E. guineensis* ground into fine powder form using a grinder. The powder was stored in polyethylene bag for extraction.

### 3.2. Extraction Procedure

Approximately 100 g of dried sample was added to 300 mL of methanol and soaked for 4 days at room temperature (30 ± 2 °C). The suspension was stirred from time to time to allow the leaf powder to fully dissolve in the methanol. Removal of the sample from solvents was done by filtration through cheesecloth and the filtrate was concentrated using a rotary evaporator in vacuo to one-fifth volume in a centrifugal evaporator at 60 °C and then sterilized by filtration using a 0.22-mm membrane for antimicrobial assay. The thick paste that obtained was further dried in an oven at 40 °C. The resultant extract (5 g extract obtained from 100 g of powdered plant material) was kept at 4 °C for further analysis.

### 3.3. Wound Healing Activity

#### 3.3.1. Crude Extract Formulation

A 10% (w/w) crude extract of the oil palm leaf was prepared by mixing 5 g of the extract in 50 g of yellow soft paraffin obtained from Pharmacy [24].

#### 3.3.2. Animal

Sixteen Sprague Dawley strain albino rats weighing between 150 to 200 g from the animal house of the AIMST University was used. The rats were placed in a room with controlled cycles of 12 h of light and 12 h of darkness; light went on at 7 am. Water and food were provided to animals ad libitum. All the rats were divided into two groups, namely the treatment group and the control. Experiments were conducted in accordance with the internationally accepted principles for laboratory animal use and care (EEC Directive of 1986; 86/609/EEC) and AIMST University Animal Use Guideline.

#### 3.3.3. *In Vivo* Wound Healing Activity

Rats were anaesthetized using 45 mg/kg of diethyl ether given by the intraperitoneal route. Full thickness wound (1.5 × 1.5 cm) was made on a shaved dorsal area. The wounds were treated topically with 10% formulated crude extract, while the control rats were treated only with yellow soft paraffin for 16 days. The decrease in wound diameters during the healing process was measured with an analytical perimeter. The wounded animals were kept for 25 days for further observations.

#### 3.3.4. Collecting of Granulation Tissue

The samplings of granulation tissues were done four times in every four days intervals (4, 8, 12 & 16 days) by using sterile scissors and forceps for histopathology studies and for analysing matrix metalloproteinases (MMPs) expression. The granulation tissues were collected without affecting other rats’ wound. This was done by collecting granulation tissues from two rats at four-day intervals. The following granulation tissues sampling was done from two new rats and not from rats previously used during the four-day interval.

#### 3.3.5. Histological Analysis

Skin tissues were collected and transferred to 10% neutral buffered formalin (NBF) for 24 h at 4 °C. The formalin fixed tissues were dehydrated through grades of alcohol and cleared in xylene and then embedded in paraffin wax (58–60° mp). Sections of 5 to 7 μm were deparaffinized and stained with hematoxylin and then counterstained with eosine [25].

#### 3.3.6. Gelatin Zymography

The presence of matrix metalloproteinases (MMPs) on the granulation tissues was analyzed by gelatin zymography [26]. One hundred milligrams (wet weight) of tissue was homogenized with Tris buffer (saline 0.9%, Tris 0.05 mg, Triton X-100 0.25%, and CaCl_2_ 0.02 M) and centrifuged at 6000 rpm for 30 min. Tissue extract was subjected to sodium dodecyl sulfate-polyacrylamide gel electrophoresis (SDS-PAGE) on 10% polyacrylamide containing 0.1% gelatin under nonreducing conditions without prior boiling. After electrophoresis, the gel was washed in 1.5% Triton X-100 for 1 h and subsequently immersed in buffer containing Tris-HCl 50 mM (pH 7.5), 1% Triton X-100, CaCl_2_ 10 mM, and 0.02% sodium azide for 16 h at 37 °C. The gel was washed several times with distilled water and stained with 0.25% Coomassie brilliant blue R250/40% methanol/10% acetic acid and destained in 7% acetic acid. Enzymatic activity was detected as clear bands of gelatin lysis against blue background. The molecular weight was determined by parallel run of samples in SDS-PAGE using standard protein markers (Genei Pvt. Ltd., Bangalore, India).

#### 3.3.7. Statistical Analysis

All results were expressed as mean ± S.D. and the results were compared statistically by One-Way Anova Test using SPSS software (student version 14.00). The *P* value < 0.05 was considered statistically significant.

## 4. Conclusions

In conclusion, Ointment from the leaf of *E. guineensis* exhibited significant pro-healing activity in the infected wound when topically applied on rats by affecting various stages of healing process. Apart from this, other properties such as antioxidant, antibacterial and antifungal activities make it a potential natural product-based ointment. The result of the present study offers pharmacological evidence to support the folkloric use of *E. guineensis* leaf for the healing of wounds in several African countries.
