# Correction: Sasidharan et al. Wound Healing Activity of *Elaeis guineensis* Leaf Extract Ointment. *Int. J. Mol. Sci.* 2012, *13*, 336–347

**DOI:** 10.3390/ijms26041441

**Published:** 2025-02-08

**Authors:** Sreenivasan Sasidharan, Selvarasoo Logeswaran, Lachimanan Yoga Latha

**Affiliations:** 1 Institute for Research in Molecular Medicine (INFORMM), Universiti Sains Malaysia, 11800 USM, Pulau Pinang, Malaysia; 2 Department of Biotechnology, Faculty of Applied Sciences, Asian Institute of Medicine, Science and Technology, 08000 Sungai Petani, Kedah, Malaysia

## Error in Figure

In the original publication [1], an error was present in Figure 2 as published. The original Figure 2 was intended to illustrate qualitative data and serve as a pictorial representation of wound-healing activity, intended more as an illustrative indicator than a definitive quantitative measure of the experimental outcome. The error occurred inadvertently during figure assembly by the student author: the corrected version of Figure 2 (Day 8) is provided below. 

The authors state that the scientific conclusions are unaffected. This correction was approved by the Academic Editor. The original publication has also been updated.

## Figures and Tables

**Figure 2:**
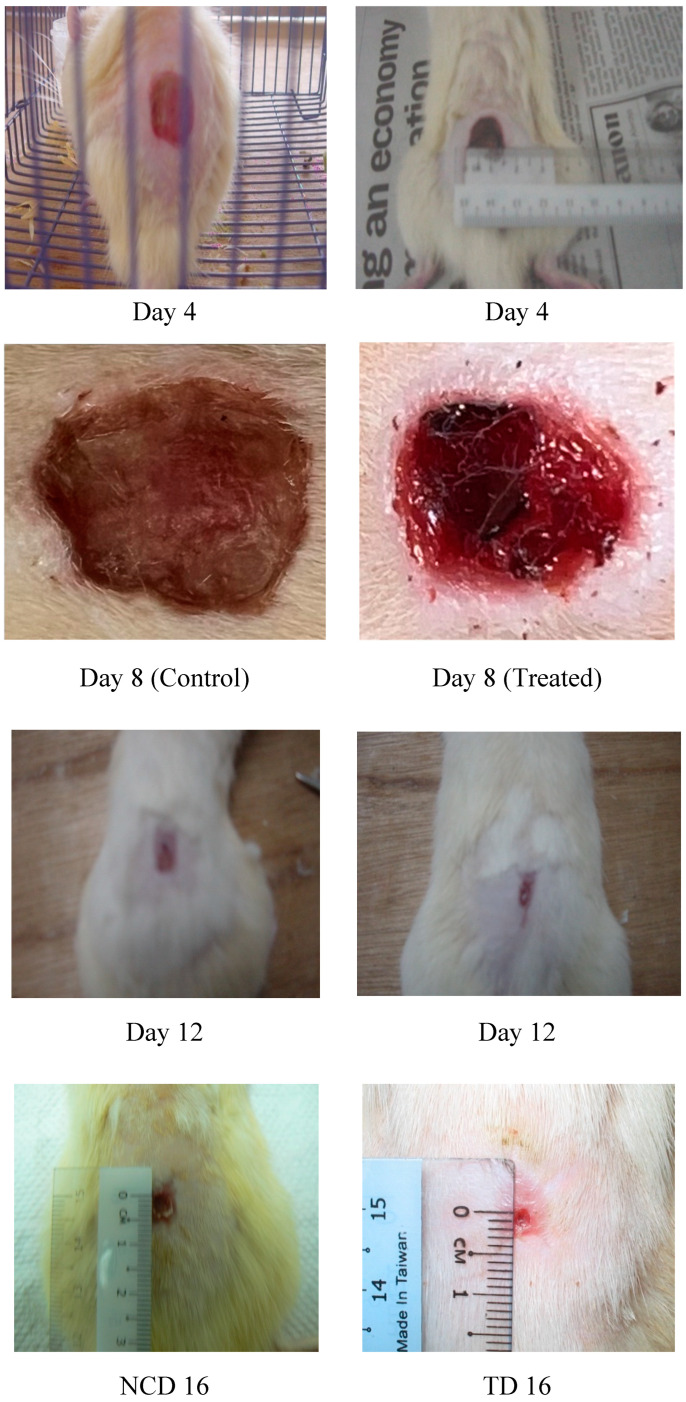
Photographical representation of contraction rate on different days in control group.
